# Architecture of the SARS-CoV-2-specific T cell repertoire

**DOI:** 10.3389/fimmu.2023.1070077

**Published:** 2023-03-20

**Authors:** Ksenia V. Zornikova, Saveliy A. Sheetikov, Alexander Yu Rusinov, Rustam N. Iskhakov, Apollinariya V. Bogolyubova

**Affiliations:** ^1^ Laboratory of Transplantation Immunology, National Medical Research Center for Hematology, Moscow, Russia; ^2^ Faculty of Biology, Lomonosov Moscow State University, Moscow, Russia

**Keywords:** T cell receptors, T cell immune response, SARS-CoV-2, T cell repertoire, T cell repertoire dynamic

## Abstract

The T cell response plays an indispensable role in the early control and successful clearance of SARS-CoV-2 infection. However, several important questions remain about the role of cellular immunity in COVID-19, including the shape and composition of disease-specific T cell repertoires across convalescent patients and vaccinated individuals, and how pre-existing T cell responses to other pathogens—in particular, common cold coronaviruses—impact susceptibility to SARS-CoV-2 infection and the subsequent course of disease. This review focuses on how the repertoire of T cell receptors (TCR) is shaped by natural infection and vaccination over time. We also summarize current knowledge regarding cross-reactive T cell responses and their protective role, and examine the implications of TCR repertoire diversity and cross-reactivity with regard to the design of vaccines that confer broader protection against SARS-CoV-2 variants.

## Graphical Abstract

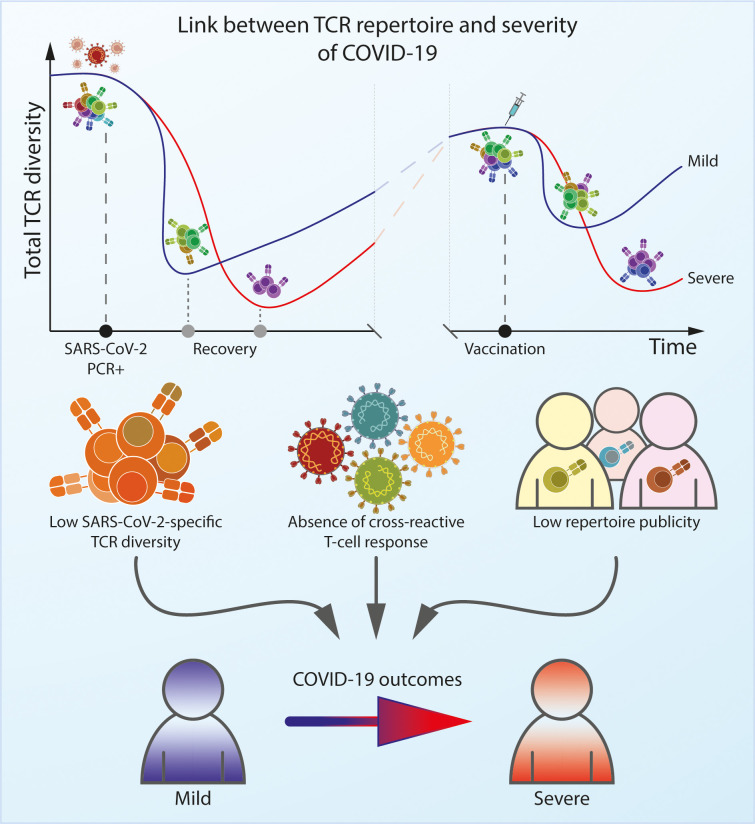

## 1 Introduction

The clinical manifestations and subsequent immune response to SARS-CoV-2 infection are diverse, with patients exhibiting a wide range of disease severity and susceptibility to future reinfections. T cells are crucial for early control and successful clearance of viral infections alongside the humoral response. The involvement of CD4^+^ and CD8^+^ T cells in the immune response reduces the severity of disease (1–3), and the presence of pre-existing SARS-CoV-2-specific T cells can prevent the development of COVID-19 (4, 5) and decrease the risk of reinfection (6). Accordingly, it has been shown that a subgroup of seronegative patients was partially protected from infection by T cells (7, 8). On the other hand, anergy of T cells is associated with a poor prognosis (9).

However, it is not only the magnitude of the T cell response but also its diversity that ultimately influences the outcome of infection (10, 11). Recently, researchers have focused on analyzing the dynamics of the TCR repertoire as an indicator of the immune response in autoimmune diseases such as multiple sclerosis (12) and rheumatoid arthritis (10), viral infections (13), and cancer (14). TCR repertoire analysis is also proving useful as a biomarker of the response to immunotherapy (15). The TCR repertoire can provide insights into immunodominance, functionality, and the protective effects of the T cell response (16, 17). Even though some conclusions may be ambiguous due to different approaches and consideration of both antigen-specific and non-antigen-specific data such as characteristics of overall TCR repertoire, TCR repertoire analysis offers a valuable tool for understanding the parameters of T cell-mediated immune responses to SARS-CoV-2 and the impact of viral mutations on immunological protection against newly-emerging SARS-CoV-2 variants.

## 2 Structure of the T cell repertoire and COVID-19 infection

The ability of the adaptive immune system to protect host organisms from a wide variety of pathogens is facilitated by the production of T cells that collectively express a large and diverse repertoire of unique TCRs. Naïve TCR diversity is generated by random rearrangement of the V and J segments of the TCR alpha (TCRα) genes and V, D, and J segments of the TCR beta (TCRβ) genes in maturing T cells within the thymus. But the ultimate structure of memory repertoire is shaped by interactions of these naïve cells with various pathogens over the course of a lifetime. The size, frequency, and publicity of individual clonotypes within a TCR repertoire can reveal both successful and failed immune responses, and recent studies have shown that the SARS-CoV-2-specific repertoire not only has its own architecture (18), but also differs depending on the severity of the disease and can change over time (19).

The SARS-CoV-2 proteome comprises at least 29 proteins (20), and as such, the number of potential epitopes is huge. However, the immunogenic regions of this proteome are unevenly distributed. ORF1 is the largest SARS-CoV-2 protein, and makes the largest contribution to T cell recognition, although the much-smaller ORF3 and Spike (S) proteins have a higher density of immunogenic epitopes compared to ORF1 (21). About 25% of the overall antigen-specific T cell response is accounted for S protein response (22). Other structural proteins are highly recognized as well and accounted for roughly 55% of CD4+ and CD8+ T cell response (22, 23). Many immunogenic epitopes in SARS-CoV-2 have been identified (24–27). However, most of these do not achieve 100% immunogenicity in convalescent donors (5) and T cells of each individual recognize 30 to 40 different CD4+ and CD8+ epitopes (22). This can be explained by the fact that the structure of the TCR repertoire is mostly determined by the presence of specific HLA alleles (28, 29), as well as the fact that epitopes compete for antigen presentation (30). Moreover, different TCRs have different probabilities of formation during the recombination process, such that the frequency of naïve cells with such TCRs may vary (28, 31). Lastly, methods of assessing immunogenicity can differ in their sensitivity and specificity, and the structure of the repertoire may vary in different studies. One particularly important factor is the time of sampling: at the peak of infection, more than 10% of total CD8^+^ T cells may be specific to a single SARS-CoV-2 epitope (21, 26). A month after infection, the frequency of most epitope-specific T cell populations is typically < 1% of total CD8^+^ T cells (24, 32–35).

The abundance of a given memory T cell clonotype in blood does not correlate with the immunodominance of its corresponding epitope (32, 35, 36). For example, studies have shown that CD8^+^ T cells specific to the highly immunogenic epitope YLQ are present at very low levels in the blood of convalescent donors (36, 37).

Limited diversity of T cell repertoire seems to be associated with severe disease (38, 39), whereas higher diversity is more likely to result in successful elimination of the virus. Multiple studies have found that the overall diversity of non-antigen-specific TCRs in blood samples taken from patients with COVID-19 is lower than that of healthy donors (40, 41), and is even lower in patients with severe disease. For example, a cohort of patients with pneumonia had a slightly less diverse overall TCR repertoire compared to those with mild disease (38) presumably due to expansion of SARS-CoV-2 specific T to defend against the infection cells in symptomatic and hospitalized individuals (42) On the other hand, low repertoire diversity may be a prognostic factor and explain the higher risk of serious illness and death in elderly patients (43–47), as it is well known that TCR repertoire diversity declines with aging, and this is also known to affect the antiviral response to other pathogens, such as the human influenza A virus (48, 49).

In general, peripheral selection and expansion of antigen-specific clonotypes driven by persistent pathogens leads to a higher proportion of shared clones among abundant clonotypes (50). The overlap of the overall non-antigen specific TCR repertoire between individuals is significantly higher in COVID-19 patients than in healthy individuals (41), and this is primarily because some epitopes of SARS-CoV-2 tend to give rise to shared, public clonotypes (35, 36, 51). Public clonotypes tend to have short CDR3 regions and arise from specific V(D)J-rearrangement events that occur with higher probability (28). Such clonotypes are thought to play a crucial role in establishing an effective pathogen-specific response and infection control of other pathogens like Cytomegalovirus (CMV), Epstein-Barr virus (EBV) and Adenovirus (52, 53).

Numerous studies have shown that the TCR repertoire in patients with mild COVID-19 infection remains relatively diverse within CDR3 central region, with high generation probability compare to severe patients (38). This leads to a broad range of SARS-CoV-2-specific sequences observed in mild disease, with many public CDR3 sequences (19, 36, 38, 54) This potentially explains why pneumonia patients have TCRs with longer CDR3 regions arising from lower-probability V(D)J-rearrangement events relative to the SARS-CoV-2-associated TCR repertoires in patients with mild disease, which also tend to prominently feature public clonotypes (38, 40). TCR repertoire profiles in asymptomatic infection is similar to mild disease (36).

It was demonstrated that preferential usage of V-, D-, and J- genes is significantly different in different viral infections including COVID-19 (55). Moreover, few studies demonstrated overrepresentation or underrepresentation of particular V-, D- and J-segments in patients with different COVID-19 clinical picture (55). Asymptomatic patients had overrepresentation of TRAV (TRAV17, TRAV12-1, TRAV19, TRAV35, and TRAV41), TRBV (TRBV12-5 and TRBV19) (56), TRAJ16 and TRBJ2-1 (57) genes compared to patients with symptoms. Frequency of TRAV2, TRAJ8, TRAJ40, TRBV3-1 and TRBV5-1 were the highest in symptomatic patients (57) while four TCBV gene segments (TRBV5-6, TRBV14, TRBV13 and TRBV24-1) were found to be overrepresented in severe patients with little Jβ gene-segment skewing (58). Another 25 sequences of the central part of the CDR3 region were found predictive for severe infection (54). The data suggest distinct clonal expansion influence on the disease progression.

## 3 Dynamics of the TCR repertoire after COVID-19 infection

During SARS-CoV-2 infection, the diversity and clonality of the antigen-specific TCR repertoire peaks within 8–14 days, then contracts slightly (25, 59) before returning to basal levels within one week after virus elimination (60). A SARS-CoV-2 specific TCR repertoire can be detected in the vast majority of convalescent patients, persisting for up to 15 months after viral clearance with a slight decrease (35, 42, 59) or even increase of clonal diversity (61). Moreover, SARS-CoV-2 epitope-specific T cells are able to proliferate in individuals who were vaccinated after infection (42) or in the re-detectable positive cases (Y. 62). Notably, SARS-CoV-1 specific T cells have demonstrated an impressive ability to persist for long periods of time, with one study detecting such clones up to 17 years after infection (63).

The durability of an antigen-specific response is determined by characteristics such as the publicity, diversity, and clonality of clonotypes recognizing that antigen (35, 51). It has been shown that long-term immunity is principally mediated by the clonal diversity of the antigen-specific T cell response (35, 61), whereas clonality does not appear to play a significant role (35). In some cases, however, dominant clones in the acute phase coincide with those found in the recovery phase (59). Numerous studies offer clear evidence that a highly diverse repertoire protects against a wide range of antigens of CMV, EBV and Human Immunodeficiency Virus-1 (HIV-1) (64, 65), and it is quite likely that such repertoires are associated with a higher level of avidity, affinity, and overall functionality (66, 67).

Despite numerous attempts to predict the longevity of virus-specific T cell immune response based on repertoire characteristics (35, 51, 68, 69), Bensouda Koraichi et al. study surmised that TCR clonotypes dynamic can be described by geometric Brownian motion. The model includes random unstimulated T cell proliferation and death, as well as asymptomatic or weakly symptomatic antigenic stimulation. However, the actual longevity of response varies from individual to individual, and in young individuals, the repertoire changes faster than in older individuals (70). Thus, at the moment, TCR clonotypes cannot be considered as the sole reliable predictor of the strength and effectiveness of the immune response.

## 4 Vaccine-induced T cell response and TCR repertoire

The high levels of mortality and morbidity associated with COVID-19 have prompted a massive, global vaccine development effort. At the time of writing this review, more than 170 vaccines have been developed, according to the World Health Organization (WHO) (https://www.who.int/publications/m/item/draft-landscape-of-covid-19-candidate-vaccines). Nearly a dozen of these are now in clinical use, and most demonstrate high efficiency in terms of protection (71, 72) and induce an immune response closely resembling that induced by infection in terms of immunophenotype, magnitude of CD4^+^ response and antibody levels (73–75). However, vaccine-induced CD8^+^ T cell expansion seems to be relatively weaker and with fewer distinct clonotype clusters compared to those induced by natural infection (76).

Only a small subset of vaccines consists of inactivated viral particles or mixtures of different viral proteins. Instead, the vast majority are aimed at inducing an immune response to the S protein (77). This approach produces a skewed T cell response that is enhanced against immunodominant epitopes (51) while also being targeted at less-dominant S-derived epitopes in vaccine recipients compared to convalescent individuals (78). In the aftermath of natural infection, the resulting CD8^+^ T cell clones are likely to recognize a broader set of viral epitopes that are not encountered in vaccines (76), and this T cell repertoire also demonstrates a higher rate of cross-recognition of epitopes from common-cold coronaviruses (79). Nevertheless, the repertoire induced by S protein-based vaccines is generally capable of protecting against existing variants as well as emerging variants of concern (VOCs) (80–82).

The antigen-specific TCR repertoire induced by both the virus and vaccines undergoes significant clonal contraction over time (79), along with an overall decrease in immune response (35), and the only way to increase protection over the long term may be booster vaccination (78).

## 5 Changes in the previously primed TCR repertoire after vaccination

Over time, SARS-CoV-2-primed T cells transition to a memory phenotype, and the diversity of the SARS-CoV-2-specific TCR repertoire decreases alongside the humoral response in convalescent individuals (35). Since T cells and antibodies provide effective antiviral protection, the exhaustion of any of them leads to a decrease in protection properties, which was shown in the large-scale prospective study (8). However, the existence of a pool of memory cells is important for fending off the virus in future encounters, and the complete absence of SARS-CoV-2-specific antibodies and T cells may lead to reinfection, although neutralizing antibodies play more important role in protection from reinfection (83)

Vaccination offers a way to boost previously primed immunity, and it has been shown that the vaccine-induced cellular response is more robust in convalescent donors. Most convalescent individuals demonstrate the same level of T cell and humoral response as previously-unexposed individuals after one shot of mRNA vaccine (84). Because the antigen-specific CD8^+^ T cell response develops more slowly than the CD4^+^ T cell response after natural infection and primary vaccination (5), it reaches its maximum only after administration of the second vaccine dose (84).

Vaccine response patterns may differ due to the difficulty of involving naïve CD8^+^ precursors in the immune response. While the vaccine-induced response of CD4^+^ T cells includes both the recruitment of memory cells and the proliferation of new, unique S protein-specific CD4^+^ T cells (79) in convalescent individuals, a rapid boost of S protein-specific CD8^+^ T cells is predominantly provided by persisting early memory S protein-specific CD38^-^CD8^+^ T cells (85, 86). Moreover, the overall magnitude of the S protein-specific CD8^+^ T cell response to vaccination in convalescent individuals is the same as in previously unexposed individuals due to the involvement of the memory compartment. However, it has been shown that vaccination selectively stimulates the expansion of S protein-specific clones and the contraction of clonotypes with non-S-protein specificity in convalescent donors (51).

SARS-CoV-2 mutations can reduce recognition of the virus by the CD8^+^ T cell compartment, possibly due to escape from HLA binding (87), although the T cell response is generally capable of effectively responding to mutant viral strains (88, 89). Vaccine-induced T cell response was also preserved across different SARS-CoV-2 variants while B cell and neutralizing antibodies recognition was significantly reduced (90). Moreover T cell response may be enhanced with booster vaccination (78, 91). which substantially increase effectiveness of protection against reinfection from 24.7% with previous infection up to 41.8% with combination of infection and vaccination. However, the most important thing is that vaccination after infection is much more effective against hospital admission or severe disease than infection alone: the effectiveness of protection increased from 74.6% to 97.4% with vaccination (92).

Nevertheless, there remains a need for further vaccine optimization and the incorporation of more immunogenic epitopes (93) that can elicit more broadly protective T cell responses (81) even in the face of the emergence of new SARS-CoV-2 variants. This is especially important for the protection of immunocompromised individuals and elderly people, and despite a greater proportion of pre-existing memory T cells in the elderly compared to the young, booster vaccination has been shown to be less effective in older individuals due to the minimal contribution of memory clonotypes in supporting high-quality T cell responses (94).

## 6 Cross-reactiveness of T cell repertoire

A more robust T cell response is also conferred by their capacity for cross-reactivity. A single T cell can cross-react to up to 10^6^–10^7^ foreign peptides (95), and this has been shown to be an essential feature of the T cell response (96–98). For some individuals who remain asymptomatic and seronegative even after close contact with COVID-19 patients (5), it has been shown that T cell-mediated protection may arise from cross-reactivity to T cells that target self-antigens and epitopes derived from various other pathogens including CMV, influenza A, EBV and HIV-1 (99, 100). Some parts of SARS-CoV-2 are very highly conserved relative to other ‘common cold’ human coronaviruses (HCoVs) (101, 102), and pre-existing protective T cells most likely originate from memory T cells derived after exposure to viruses such as HCoV-OC43, HCoV-HKU1, HCoV-NL63 and HCoV-229E (4), which circulate widely in the human population (63, 103, 104).

Some studies have shown that more than 20% of pre-pandemic samples contained SARS-CoV-2-reactive T cells (63, 103, 104), which protect patients from developing severe illness (105). But other research has failed to confirm such strong cross-reactivity, and has instead revealed that these cross-reactive T cells from pre-pandemic samples have a predominantly naïve phenotype, which means that they did not develop from an immune response to HCoVs (32, 77). This difference in results may be attributable to the choice of peptides used in the study. T cells specific to peptides that are conserved among coronaviruses are more abundant and tend to have a memory phenotype compared to those which recognize unique SARS-CoV-2 peptides. Notably, CD8^+^ T cells that cross-react to these conserved epitopes are much more plentiful in patients with mild COVID-19 versus those with severe illness, suggesting a protective role (105). Moreover, TCR repertoires that recognize the same conserved peptides were similar in unexposed donors and convalescent individuals (106). Other studies have suggested that pre-existing T cells that react to SARS-CoV-2 RNA polymerase may also be associated with asymptomatic disease (4, 107).

One of the most cross-reactive epitopes in unexposed individuals is SPRWYFYYL_N105-113_ (SPR) restricted in HLA-B*07:02 (77, 108). The immunodominance of SPR originates from a high frequency of naïve precursors in pre-pandemic samples. Many naïve SPR progenitors arise from a highly diverse TCRαß repertoire (77, 109), and a diverse SPR-specific CD8^+^ T cell response with high functional avidity and antiviral effector functions has been detected in patients with mild disease compared to individuals with severe COVID-19 (108). Interestingly to note that only one SPR homologous epitope from HCoVs, LPRWYFYYL, has also demonstrated the ability to elicit a cross-reactive response (109). Other highly immunodominant epitopes, like KPRQKRTAT_N257-265_ (KPR), YLQPRTFLL_S269-277_ (YLQ), and QYIKWPWYI_S1208-1217_ (QYI), have been shown to be abundant in pre-existing naïve T cell repertoires (32, 77, 108).

The importance of cross-reactive T cell response for protection against newly-emerging mutant strains is well established (110). New VOCs may be less susceptible to neutralizing antibodies (80, 111, 112), while T cells retain their protective capabilities (89, 113, 114). This protective capacity is shaped by the wide variety of epitopes recognized in different people (21). Nevertheless, the emergence of non-synonymous mutations in some T cell epitopes can lead to a decrease in peptide and MHC binding or a reduced ability to activate T cells (115, 116). However, such mutations are rarely found in VOCs, and it is likely that an epitope that evades presentation by one HLA allele will become presentable by the other (87). This mechanism may explain why the magnitude of T cell response to new variants is typically decreased by only 20–30% (89, 117).

It should also be noted that cross-recognition does not always provide protection, and can also be associated with worse disease outcomes; this suggests that other mechanisms are coming into play, including age-related differences in the involvement of different cell populations in the immune response (118). Several studies have examined the potential protective effects of Bacille Calmette–Guérin (BCG) immunization due to the presence of epitopes that resemble epitopes from SARS-CoV-2 (119, 120), but clinical trials have offered no evidence for such protection (121).

## 7 Conclusion

The severity of COVID-19 can vary from asymptomatic to lethal disease, and many different factors contribute to the outcome of infection, most notably including gender, ethnicity, health, and age (46, 47). However, demographics only partially explain the differences in mortality rates between countries (122), and numerous studies strongly point to the influence of the TCR repertoire on the ultimate course of infection. It has been shown that TCR repertoire diversity and clonality might determine the success of the immune response to both the virus and the vaccine, and several machine learning-based tools have been developed and applied in order to distinguish between convalescent and naive individuals (18) and predict disease outcomes (19, 54, 123) based on TCR repertoire. Despite high hopes and numerous studies of cross-reactive responses from pre-existing immunity to other HCoVs and other pathogens, protectiveness of cross-recognition is still debating (124). The studies to date have shown that protectiveness of cross-recognition in the context of prior infection with SARS-CoV-2 against reinfection is relatively low and waned to 24·7% at 12 months, but may be significantly improved with vaccination (92). Against this backdrop, the ongoing spread of SARS-CoV-2 and emergence of new, potentially immune-escaping VOCs reinforces the urgency of further optimizing the composition of vaccines based on insights derived from research into the T cell response against SARS-CoV-2.

## Author contributions

AB, and KZ contributed to conception of the review. KZ wrote the first draft of the manuscript. KZ, SS, AR, and RI wrote sections of the manuscript. SS drew the graphical abstract. All authors contributed to manuscript revision, read, and approved the submitted version. All authors contributed to the article and approved the submitted version.
